# Underweight in Young Adult Women as a Dynamic Nutritional State: Evidence from Four Complementary Longitudinal Methods

**DOI:** 10.3390/nu18071156

**Published:** 2026-04-03

**Authors:** Katsumi Iizuka, Hitomi Matsuura, Kotone Yanagi, Eri Hiraiwa, Yuka Sato, Kiyomi Kaito, Risako Yamamoto-Wada, Kanako Deguchi, Hiroyuki Naruse

**Affiliations:** 1 Department of Clinical Nutrition, School of Medicine, Fujita Health University, Toyoake 470-1192, Japan; 51023103@fujita-hu.ac.jp (H.M.); 51022099@fujita-hu.ac.jp (E.H.); 51023051@fujita-hu.ac.jp (Y.S.); risako.wada@fujita-hu.ac.jp (R.Y.-W.); kanasakuran@gmail.com (K.D.); 2 Faculty of Medicine, Fujita Health University, Toyoake 470-1192, Japan; 3 Health Management Center, Fujita Health University, Toyoake 470-1192, Japan; yanagi-k@fujita-hu.ac.jp (K.Y.); kkaito@fujita-hu.ac.jp (K.K.); hnaruse@fujita-hu.ac.jp (H.N.)

**Keywords:** underweight, young underweight women, dynamic nutritional state, boundary-crossing phenotypes

## Abstract

Background: Underweight (BMI < 18.5 kg/m^2^) remains prevalent among young Japanese women but lacks standardized measurement approaches. We compared four analytical methods and identified discrepancies. Methods: A retrospective analysis of 883 underweight women aged 20–29 years followed for 6.1 ± 4.2 years was performed. We compared (1) year-to-year transitions, (2) state occupancy, (3) the Aalen–Johansen estimator, and (4) Kaplan–Meier Survival Analysis. We performed bidirectional flow analysis quantifying inflow/outflow rates, BMI distribution analysis, and time-weighted classification. Results: Methods 1 and 4 showed 31-point discrepancies (78.1% vs. 47.1% in women). In bidirectional flow, inflow exceeded outflow at ages 22–27 (35.7%/yr vs. 20.7%/yr, outflow/inflow ratio: 0.58), balanced at ages 27–37 (ratio: 1.02) and showed outflow-dominant pattern at ages 37–47 (ratio: 4.92). BMI clustered at 18.0–19.0 kg/m^2^ (42.7%); 69.4% crossed the threshold once. Time-weighted classification revealed four phenotypes: persistent (≥75% time underweight; 40.1%, BMI: 17.54 kg/m^2^), moderate (50–74%; 17.6%, BMI: 18.40 kg/m^2^), intermittent (25–49%; 17.6%, BMI: 18.97 kg/m^2^), and transient (<25%; 24.8%, BMI: 19.49 kg/m^2^). The moderate + intermittent group showed yo-yo phenotypes (35.2%). Conclusions: Underweight in young Japanese women should be viewed as a heterogeneous dynamic nutritional state. The methodological discrepancy, threshold crossing, and phenotypic classification show that BMI-defined underweight comprises distinct patterns. Cross-sectional data evaluation may lead to incorrect assessments. Future research examining relationships between longitudinal low body weight subgroups and clinical outcomes could identify at-risk populations within the underweight group.

## 1. Introduction

An underweight status, defined as a body mass index (BMI) < 18.5 kg/m^2^, remains a significant public health concern in Japan, particularly among young women [1,2,3,4]. According to the National Health and Nutrition Survey, the prevalence of underweight has persistently exceeded 20% among women aged 20–29 years over the past two decades, which is substantially higher than the value reported in other developed countries [5]. This trend contrasts sharply with the global obesity epidemic and raises concerns about long-term health consequences specific to this population [6].

Chronic underweight is associated with increased risks of osteoporosis and fragility fractures in middle-aged and older individuals, adverse pregnancy outcomes, and increased mortality [7,8,9]. Specifically, a low BMI during young adulthood is a critical risk factor for a reduced bone mineral density, which manifests clinically as osteoporotic fractures in postmenopausal women [10]. Additionally, an underweight status has been linked to sarcopenia, immune dysfunction, and reduced quality of life in recent years [11,12,13]. Our previous studies revealed distinct metabolic and nutritional profiles in young underweight women [3,14,15]. We demonstrated that nearly all underweight women exhibit vitamin D deficiency, with 20% also having vitamin B_12_ and folate deficiencies [3]. Notably, vitamin B_1_ deficiency is more prevalent in underweight women aged 40 years and older than in those aged 20–39 years, suggesting the occurrence of age-related metabolic modifications [3,14]. Furthermore, we found that body fat percentage and the skeletal muscle index are independently associated with the lymphocyte count and grip strength, respectively, regardless of BMI [14]. Most recently, we reported that, compared with normal-weight women, underweight women exhibit a reduced gut microbiota diversity despite having a similar dietary diversity [15]. These findings indicate that compared with normal-weight individuals, underweight women have unique metabolic dynamics, which may be further modified by age.

Despite the well-documented prevalence and health risks, critical gaps remain in our understanding of the longitudinal trajectories of BMI among young underweight women. Only one study has compared changes in body weight from birth to ages 12, 15, and 20, as well as current body composition and dietary intake among young underweight Japanese women, reporting that underweight women consistently follow a lower weight trajectory from birth through childhood and adolescence [16,17]. Specifically, what proportion of young underweight women maintain this status at middle and older ages, how frequently transitions between underweight and normal weight occur over extended follow-up, and whether identifiable subgroups with distinct persistence patterns exist remain unclear. Most existing studies have relied on cross-sectional data or short-term follow-up, limiting the ability to characterize long-term BMI dynamics and distinguish transient underweight from chronic, persistent underweight. Furthermore, the clinical and epidemiological definitions of underweight persistence lack consensuses, with varying approaches used to measure persistence over time.

Our observations on dietary and gut microbiota patterns raised questions about the appropriateness of the current BMI threshold (BMI < 18.5 kg/m^2^) for defining underweight. We observed that dietary diversity is optimal at a BMI of 18.0–19.0 kg/m^2^ and that gut microbiota diversity increases linearly from a BMI of 16 to 19 kg/m^2^ before plateauing. These findings suggest that the conventional threshold may not adequately capture clinically significant chronic underweight. However, these findings are exploratory and should not be interpreted as direct evidence for redefining the BMI cutoff. Rather, they suggest the possibility that young thin women comprise distinct subgroups with different subsequent BMI trajectories, warranting longitudinal investigation.

We conducted a retrospective longitudinal cohort study using health examination data spanning more than two decades to address these gaps. Our primary objectives were (1) to compare four distinct analytical methods for assessing underweight persistence and elucidate discrepancies in short-term versus long-term persistence estimates; (2) to quantify BMI clustering and dynamic fluctuation patterns around the conventional diagnostic threshold (BMI 18.5 kg/m^2^); (3) to classify individuals based on time-weighted persistence and characterize subgroups with varying degrees of long-term underweight exposure; and (4) to examine whether these trajectory-based patterns can help refine the characterization of chronic underweight. By integrating multiple analytical approaches, this study aimed to provide a comprehensive description of underweight dynamics in young women. Because clinical outcomes were not directly assessed, our findings are intended to be hypothesis-generating and to provide a foundation for future studies evaluating whether distinct BMI trajectories are associated with clinically meaningful health consequences.

## 2. Materials and Methods

### 2.1. Study Design and Participants

We conducted a retrospective longitudinal cohort study of female employees at Fujita Health University who underwent routine health examinations between April 2003 and March 2025. The study included adults aged 20–59 years with at least five health examination records during the observation period. From the full cohort (*N* = 4328 (women)), we identified 883 women who were underweight (BMI < 18.5 kg/m^2^) at least once during their twenties (aged 20–29 years). This subgroup constituted our primary analytical cohort for investigating underweight persistence patterns. The mean follow-up duration was 7.4 ± 5.0 years. The physical data and food frequency questionnaires were provided by the healthcare center at our university in a fully anonymized form, so the data were depersonalized (accessed on 6 December 2025). In this study, we analyze data that has already been anonymized, making it impossible to identify individuals. For this reason, we announced on the website of the Department of Clinical Nutrition, Fujita Medical School (the approval date: from 21 April 2025 to 31 March 2027), that exclusion is not possible. The study was conducted according to the principles of the Declaration of Helsinki and approved by the Research Ethics Committee of Fujita Health University (application numbers: HM25-356 (25 November 2025), HM25-606 (9 March 2026)).

### 2.2. Data Collection and Definitions

Health examination data included anthropometric measurements (height and weight) and demographic information (age and sex). BMI was calculated as weight in kilograms divided by height in meters squared (kg/m^2^). BMI was classified into three categories according to the World Health Organization criteria: underweight (BMI < 18.5 kg/m^2^), normal weight (18.5 ≤ BMI < 25.0 kg/m^2^), and overweight/obese (BMI ≥ 25.0 kg/m^2^). Age groups were stratified into four 10-year categories: 20s (20–29 years), 30s (30–39 years), 40s (40–49 years), and 50s (50–59 years). We defined a “threshold zone” as a BMI of 18.0–19.0 kg/m^2^, spanning ±0.5 kg/m^2^ around the diagnostic cutoff of 18.5 kg/m^2^, representing a high-risk region for frequent state transitions. A “boundary crossing” was defined as a transition between underweight and normal weight states between consecutive observations.

### 2.3. Four Analytical Methods for the Assessment of Underweight Persistence

We employed four distinct statistical approaches that differ in their conceptual focus and measurement units to comprehensively evaluate BMI state transitions.

#### 2.3.1. Method 1: Year-to-Year Transition Probability

We calculated year-to-year persistence rates by extracting consecutive annual measurement pairs with exactly 1-year intervals to assess short-term BMI state stability. For individuals classified as underweight in a given year, we determined the proportion who remained underweight in the following year. This method captures the conditional probability at 1-year intervals and reflects immediate state stability, including individuals who cross the 18.5 kg/m^2^ BMI threshold repeatedly. The analyses were stratified by age group (20s, 30s, 40s, and 50s) and sex. Ninety-five percent confidence intervals were calculated using the Wilson score method to account for binomial distribution properties.

#### 2.3.2. Method 2: Long-Term State Occupancy Probability

Individuals classified as underweight in their 20s were identified and tracked longitudinally across subsequent decades. We calculated the cross-sectional prevalence of underweight status in each age group (30s, 40s, and 50s), representing the proportion of underweight observations among multiple assessments. This approach captures the long-term burden of underweight status, including weight cycling patterns with repeated transitions. The observation-level prevalence was averaged across individuals with different follow-up patterns. Analyses were stratified by sex, and only age groups with at least 10 observations were included to ensure stable estimates. Ninety-five percent confidence intervals were calculated using the Wilson score method.

#### 2.3.3. Method 3: Aalen–Johansen Estimator (Competing Risk Model) [18]

Among individuals who were underweight in their 20s, we applied the Aalen–Johansen estimator to model the probability of continuous persistence without any transition from underweight to normal weight. We employed a two-state model (underweight ⇄ normal weight only), excluding transitions to the overweight/obese category, to isolate true continuous underweight persistence. This approach accounts for transitions to other weight categories and provides the most conservative estimate of underweight persistence by treating exit to normal weight as a competing event. Using the R packages mstate and survival, we calculated cumulative incidence functions stratified by sex and reported probabilities at the midpoint of each age group (representing average persistence during that decade). The results were not calculable for age groups with fewer than 5 at-risk individuals.

#### 2.3.4. Method 4: Kaplan–Meier Survival Analysis [19]

We performed a Kaplan–Meier survival analysis to estimate the probability of continuous underweight persistence from baseline until the first transition to normal weight. Baseline was defined as the first measurement during the 20s at which an individual was classified as underweight (BMI < 18.5 kg/m^2^). The primary event was the first transition from underweight to normal weight (BMI ≥ 18.5 kg/m^2^). Individuals without transitions were censored at their last observation or at age 60, whichever occurred first.

Individuals with no follow-up observations after baseline (time-to-event = 0) were excluded because a measurable risk interval was needed for the survival estimation. All 883 women had sufficient follow-up.

This method provides a conservative estimate of structural persistence by quantifying the duration of uninterrupted underweight. The 10-year area under the survival curve (AUC) was calculated to summarize the cumulative persistence probability within each age group. Survival curves were compared between sexes via the log-rank test [19].

We also evaluated whether discrepancies between the analytical methods were attributable to dichotomization at the 18.5 kg/m^2^ BMI threshold by conducting sensitivity analyses using alternative underweight cutoffs of 18.3 and 18.7 kg/m^2^. All primary analyses (Methods 1 and 4) were repeated with these thresholds. This approach allows for the assessment of robustness against potential boundary artifacts resulting from the dichotomization of a continuous variable.

### 2.4. Bidirectional Flow Analysis

We clarified the mechanisms underlying the discrepancies between short-term and long-term persistence estimates by quantifying age-specific bidirectional transition rates between underweight and normal weight states.

We restricted the analysis to individuals who were underweight (BMI < 18.5 kg/m^2^) at least once in their 20s and constructed 1-year consecutive observation pairs within each sex and age band (22–27, 27–37, 37–47 years).

Inflow was defined as the conditional 1-year transition probability from normal weight to underweight, *p* in = *P* (UW t + 1 ∣ Normal t), computed as *n* Normal → UW/*n* Normal.

Outflow was defined as the conditional 1-year transition probability from underweight to non-underweight, *p* out = *P* (nonUW t + 1 ∣ UW t), computed as *n* UW → nonUW/*n* UW.

We summarized bidirectional dynamics using the ratio R = *p* out/*p* in (values < 1 indicate inflow-dominant and values > 1 indicate outflow-dominant patterns).

The 95% confidence interval for R was obtained by nonparametric bootstrap resampling of 1-year transitions within each stratum (e.g., 4000 replicates) and taking the 2.5th and 97.5th percentiles.

### 2.5. Persistence-Based Classification with an Analysis of Boundary Crossing

#### BMI Threshold Clustering and Dynamic Fluctuation Analysis

We analyzed all available observations from the underweight cohort in their 20s (*n* = 883) to quantify BMI clustering around the diagnostic threshold and characterize dynamic weight fluctuation patterns. We assessed five key dimensions:

(1) BMI distribution and threshold zone concentration. We calculated the proportion of observations falling within the “threshold zone” (BMI 18.0–19.0 kg/m^2^), representing ±0.5 kg/m^2^ around the diagnostic cutoff. We also examined a narrower zone (BMI 18.25–18.75 kg/m^2^) representing ±0.25 kg/m^2^ around the cutoff. The threshold zone (18.0–19.0 kg/m^2^) was defined operationally to capture BMI fluctuation around the conventional cutoff of 18.5 kg/m^2^. This range was empirically motivated by the observed clustering of underweight BMI values around 18.0–18.5 kg/m^2^ and by the finding that annual BMI changes were within approximately ±0.5 kg/m^2^ in about half of the participants.
(2) Boundary-crossing frequency. A “boundary crossing” was defined as a transition between underweight (BMI < 18.5 kg/m^2^) and normal weight (BMI ≥ 18.5 kg/m^2^) between consecutive observations. We calculated the number of crossings per individual over the entire follow-up period. Individuals with ≥3 crossings were classified as exhibiting “recurrent fluctuation patterns”. The yo-yo pattern was defined as ≥3 crossings of the BMI 18.5 kg/m^2^ threshold in order to identify repeated transitions between underweight and normal-weight categories, rather than isolated or transient fluctuations.
(3) Annual BMI change was calculated as the absolute BMI change per year between consecutive observations using the equation annual BMI change = |BMI t + 1 − BMI t|/(Year t + 1 − Year t). Changes ≥ 0.5 kg/m^2^/year were classified as “substantial fluctuations” as this magnitude can easily facilitate boundary crossings across the 1.0 kg/m^2^ threshold zone width.
(4) Descriptive statistics. For each individual, we calculated the mean, standard deviation, minimum, and maximum BMI across all observations to characterize within-person variability.

### 2.6. Time-Weighted Persistence Classification

We quantified heterogeneity in underweight persistence patterns and identified clinically distinct subgroups by classifying individuals in the 20s underweight cohort (*n* = 883) by the proportion of follow-up time spent with a BMI <18.5 kg/m^2^. For each individual, we calculated the persistence percentage using the following equation: Persistence percentage = (number of underweight observations/total number of observations) × 100. Based on this time-weighted metric, individuals were categorized into four persistence groups: persistent (≥75%), moderate (50–74.9%), intermittent (25–49.9%), and transient (<25%). This classification captures the cumulative burden of underweight exposure over time, accounting for both continuous persistence (characteristic of the persistent group) and recurrent fluctuation patterns with repeated transitions between underweight and normal weight states (characteristic of the moderate and intermittent groups). As the basis for dividing the groups into four categories, we focused on the proportion of time spent underweight, since many individuals fluctuate between being underweight and having a normal weight. For each persistence category, we calculated (1) the mean BMI and standard deviation across all observations; (2) the BMI distribution across four zones (<18.0 kg/m^2^, 18.0–18.5 kg/m^2^, 18.5–19.0 kg/m^2^, and ≥19.0 kg/m^2^); (3) the threshold zone concentration (proportion of observations in the BMI range of 18.0–19.0 kg/m^2^); (4) the narrow zone concentration (proportion in the BMI range of 18.25–18.75 kg/m^2^); (5) the boundary-crossing frequency; and (6) the recurrent fluctuation pattern prevalence (≥3 crossings).

### 2.7. Analysis of the BMI Trajectory of Participants in the Different Persistence Categories

Mean BMI trajectories were constructed for each persistence category stratified by sex. Age-stratified mean BMI values were calculated at 5-year intervals (20–24, 25–29, 30–34, 35–39, 40–44, 45–49, and 50–54 years) with 95% confidence intervals using locally weighted scatterplot smoothing (LOESS). Linear mixed-effects models were used to test for differences in BMI trajectories across persistence categories, with random intercepts for individuals and fixed effects for age, persistence category, and their interaction. Pairwise comparisons were performed using Tukey’s honest significant difference test.

### 2.8. Analysis of State Transition Patterns

We calculated the distribution of transition types between consecutive observations (time interval: median of 1.0 years [IQR 0.8–1.2]) to characterize the dynamic nature of BMI state transitions within each persistence category. Four transition types were defined: (1) stay underweight (BMI < 18.5 → <18.5), (2) improve to normal (BMI < 18.5 → 18.5–24.9), (3) revert to underweight (BMI 18.5–24.9 → <18.5), and (4) stay normal (BMI 18.5–24.9 → 18.5–24.9). Among the persistence categories, “stay normal” represents temporary periods of normal weight within a predominantly underweight trajectory. Transition frequencies were calculated as percentages of total transitions within each persistence category, stratified by sex. Transition frequencies were calculated as percentages of total transitions within each persistence category, stratified by sex. Furthermore, to characterise the BMI distribution within each persistence category, all observations from index cohort members were pooled and assigned to one of four BMI zones: severely underweight (BMI < 18.0 kg/m^2^), near-threshold below (18.0–18.4 kg/m^2^), near-threshold above (18.5–18.9 kg/m^2^), and normal range (≥19.0 kg/m^2^). The proportion of observations falling within each zone was calculated for each persistence category and displayed as a stacked bar chart. The near-threshold zone (18.0–18.9 kg/m^2^) was defined to capture observations within ±0.5 kg/m^2^ of the WHO underweight threshold (18.5 kg/m^2^), representing the region where state transitions are most likely to occur. Since 46% of the individuals in the underweight cohort of this study fall within this range (18.0–18.9 kg/m^2^) and the change in body weight over one year is also within 0.5 kg, we focused our investigation on this area. Chi-square tests were used to compare transition distributions across persistence categories.

### 2.9. Statistical Analysis

Significant differences across persistence categories were assessed using one-way analysis of variance (ANOVA) for approximately normally distributed continuous variables and chi-square tests for categorical variables. Continuous variables with clearly non-normal or discrete distributions were analyzed using the Kruskal–Wallis test as a rank-based nonparametric alternative. Post hoc pairwise comparisons were conducted using Tukey’s honestly significant difference (HSD) test following ANOVA and Dunn’s test with Bonferroni correction following the Kruskal–Wallis test to account for multiple comparisons. All the statistical tests were two-sided, and the significance level was set at α = 0.05.

All analyses were performed using R version 4.3.0 (R Foundation for Statistical Computing, Vienna, Austria). The key packages used included dplyr (data manipulation), survival (survival analysis), mstate (multistate modeling), ggplot2 (visualization), and binom (confidence interval estimation).

### 2.10. Ethical Considerations

This study was approved by the Institutional Review Board of Fujita Health University (approval number: HM25–356). All the data were anonymized to protect participants’ privacy. The requirement for informed consent was waived because of the retrospective nature of the study, which used existing health examination records. This information was posted as a public disclosure document on the university website.

## 3. Results

### 3.1. Participant Characteristics and Follow-Up Overview

#### Baseline Cohort

A total of 4328 women were included in the analysis. The mean age at baseline was 29.5 ± 9.4 years, and the mean BMI was 21.7 ± 3.1 kg/m^2^. At baseline, 612 individuals (14.1%) were underweight (BMI < 18.5 kg/m^2^), 3394 (78.4%) had a normal weight, and 612 (14.1%) were overweight or obese. Ages were 27.8 years old and had normal BMIs (20.9 kg/m^2^). The median follow-up duration was 6.0 years (IQR: 4–11; mean: 8.5 ± 5.6 years). The detailed baseline characteristics are shown in Table 1.

**Figure 1:**
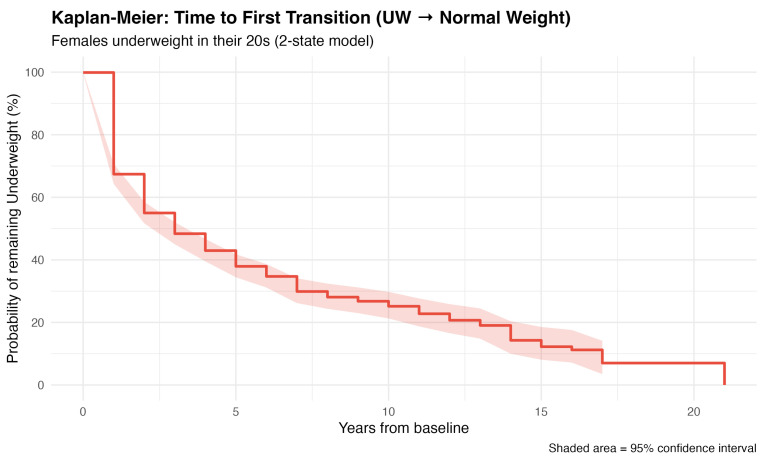
Kaplan–Meier Survival Curves: Time to First Transition from Underweight to Normal Weight in female employees.

Among the 4328 women, 883 women (20.4%) were underweight during their 20s; this group comprised the study cohort for the longitudinal analysis. This cohort had a mean age of 22.7 ± 1.9 years and a mean BMI of 18.5 ± 1.1 kg/m^2^ at baseline. The median follow-up duration for this cohort was 6 years (IQR: 4–9.0; mean: 7.4 ± 5.0 years). The baseline characteristics of the study cohort are presented in Table 2. Among the 20s underweight cohort (*n* = 883), the results of the Kaplan–Meier analysis revealed substantial heterogeneity in weight trajectories (Figure 1). While 60% of the participants transitioned to a normal weight within 5 years, a notable minority—25% of women—remained underweight for ≥10 years. The median time to first exit was 3.2 years.

**Table 2:** Baseline Underweight 20s female employee cohort.

	Level	Female
*N*		883
Age (years)		22.70 (1.89) ^a^
Height (cm)		158.19 (5.25) ^a^
BW (kg)		46.27 (3.97) ^a^
BMI (kg)		18.48 (1.14) ^a^
BMI state (%)	Normal weight	400 (45.3) ^a^
	Overweight	1 (0.1) ^a^
	Underweight	482 (54.6) ^a^
Follow-up duration (years)		7.4 (5.0) ^a^
Follow-up duration (years)		6.0 (4.0–9.0) ^b^

Note. *n* = 883 women who were underweight (BMI < 18.5 kg/m^2^) at any time during their 20s (883 females). The BMI_state distribution reflects baseline measurements: 482 females (54.6%) were underweight at their first health examination. For Method 3 (Aalen-Johansen estimator), the analysis cohort included individuals underweight at the start of their 20s (age 20–22) with sufficient follow-up data (*n* = 483 females; see Table 3). The 1-person difference (482 vs. 483) reflects individuals who became underweight during their early 20s rather than at age 20. Data presented as ^a^ mean (SD) and ^b^ median (IQR).

**Table 3:** Four Methods for Integrated Comparison of Women.

	Method 1		Method 2		Method 3		Method 4	
Age	Rate	N	Rate	N	Rate	*n*	Rate	N
20s	78.1 (76.4–79.7)	2446	59.9 (58.6–61.2)	5406	36.5 (32.4−40.9)	493	47.1 (43.1–51.1)	883
30s	75.0 (72.4–77.4)	1139	47.5 (45.3–49.7)	1963	3.7 (1.1–13.0)	52	16.6 (12.6–20.6)	50
40s	75.3 (71.5–78.7)	538	22.0 (18.5–26.0)	459	N/A	NA	0.0 (0.0–4.0)	1
50s	84.6 (79.9–88.4)	279	N/A	NA	N/A	NA	N/A	N/A

Definition. Method 1: Year-to-year maintenance, Short-term stability, Conditional probability at 1-year intervals; Method 2: Long-term recurrence in observations, Proportion of UW observations among multiple assessments; Method 3: Aalen-Johansen (midpoint), Survival under competing risks, Accounts for transitions to other weight categories; Method 4: Kaplan–Meier (10-year AUC), Time-weighted average. UW = Underweight (BMI < 18.5 kg/m^2^), *n* = sample size at baseline (20s cohort), Method 1: P(UW at t + 1 | UW at t); *N* = number of transitions, Method 2: Proportion of observations classified as UW; *N* = total observations; Method 3: Probability of remaining UW until age-group midpoint (competing risks); *n* = individuals, Method 4: Area under Kaplan–Meier curve/follow-up duration; *n* = individuals, 95% CI in parentheses. Confidence intervals calculated using the Wilson score method. Detailed time-series data available in Figure 1 and Table 3 (Kaplan–Meier curve). Note: N/A indicates insufficient sample size (<5 individuals) or no observations available for that age-method combination. Confidence intervals may be wide for small samples (indicated by overlapping error bands in the figure).

Kaplan–Meier curves showing the probability of remaining underweight over time among women who started in the underweight category during their 20s women (*n* = 883). Time zero represents the baseline observation in the 20s age group. The y-axis indicates the probability (%) of remaining underweight, and the x-axis shows years from baseline. Shaded areas represent 95% confidence intervals. The curves demonstrate a gradual transition from underweight to normal weight over time, with approximately 50% of individuals transitioning to normal weight within 5 years and 75% within 10 years. No statistically significant difference was observed between sexes (log-rank test *p* > 0.05). Participants were censored at their last observation or at age 60, whichever came first. Method 4: *n* represents individuals with ≥1 follow-up observation after baseline measurement.

### 3.2. Comparison of Low Body Weight Persistence Rates Using Four Short-Term and Long-Term Evaluations

**Figure 2:**
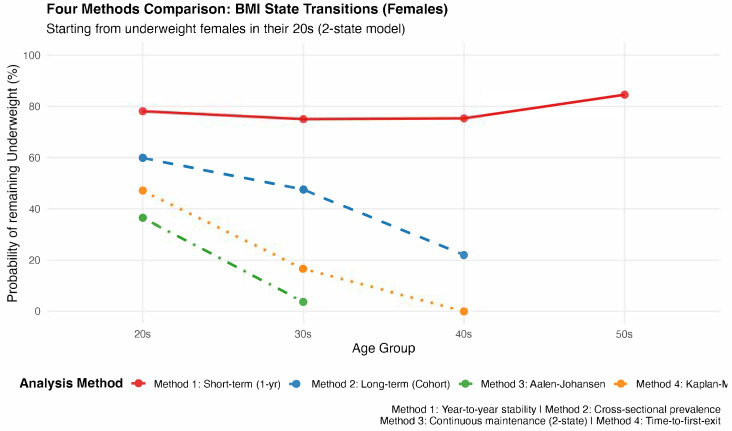
Comparison of Four Methods: Underweight Maintenance Probability Using Different Analytical Approaches. Comparison of underweight maintenance probability estimates using four different analytical methods, each with distinct definitions and sample sizes. The x-axis shows age groups (20s, 30s, 40s, 50s) and the y-axis shows probability (%) with 95% confidence intervals. Our analytical methods are compared: Method 1 (red solid line)—Year-to-year maintenance (Short-term stability): Conditional probability of remaining in the same BMI category between consecutive annual observations. Based on observation pairs (e.g., Female 20s: *N* = 2446 pairs). The x-axis shows age groups (20s, 30s, 40s, 50s) and the y-axis shows probability (%). This method captures short-term stability. Method 2 (blue dashed line)—Long-term recurrence in observations: Proportion of underweight observations among multiple assessments within each age decade. Based on total observations (e.g., Female 20s: *N* = 5406 observations). Method 3 (green dash-dot line)—Aalen-Johansen estimator (2-state model): Probability of continuous underweight maintenance without any transition to normal weight, accounting for competing risks and time-dependent transitions. Based on individuals entering each age group while still underweight (e.g., Female 20s: *n* = 493). Not calculable for older age groups due to insufficient sample size (most individuals had transitioned to normal weight). Method 4 (orange dotted line)—Kaplan–Meier time-to-first-exit (10-year AUC): Time-weighted average probability of remaining underweight, calculated as area under the Kaplan–Meier survival curve over 10 years within each age decade. Based on individuals starting each decade (e.g., Female 20s: *N* = 883). N represents individuals with ≥1 follow-up observation after baseline measurement. Six males were excluded from the original cohort (112 → 106) due to absence of follow-up data within their 20s (time-to-event = 0, not estimable in survival analysis).

The baseline cohort (*n* = 4328) was predominantly young (27.84 ± 8.84) years, with a mean BMI in the normal range (Table 1). We compared four methods across age groups to assess underweight persistence (Figure 2 and Table 3).

The four methods differed in their conceptual focus: Method 1 assessed short-term stability (year-to-year transitions), Method 2 measured observation-level prevalence, Method 3 modeled competing risk-adjusted persistence, and Method 4 quantified time-weighted exposure.

Regarding the short-term persistence of underweight status (Method 1), the 1-year persistence rate of underweight status among women ranged from 75.0% to 84.6% across age groups. Specifically, the persistence rates were 78.1% (95% CI: 76.4–79.7%) for women in their 20s (*n* = 2446 transitions), 75.0% (72.4–77.4%) for women in their 30s (*n* = 1139), 75.3% (71.5–78.7%) for women in their 40s (*n* = 538), and 84.6% (79.9–88.4%) for women in their 50s (*n* = 279). (Table 3).

With respect to long-term state occupancy probability (Method 2), for women (*n* = 1802 individuals contributing 7828 total measurements), the proportion of examinations classified as underweight decreased progressively with age: 59.9% (95% CI: 58.6–61.2%) of 5406 measurements in 20s, 47.5% (45.3–49.7%) of 1963 measurements in 30s, and 22.0% (18.5–26.0%) of 459 measurements in 40s (Table 3).

According to the results from the Aalen–Johansen estimator, i.e., the continuous persistence probability (Method 3), among women who were first underweight in their 20s, the probability of continuous persistence decreased rapidly with time. By the midpoint of their 20s (~age 24–25), 36.5% (95% CI: 32.2–40.8%) of the participants were underweight without any transition to normal weight. This percentage decreased sharply to 9.3% (95% CI: 7.5–11.5%) by the age of 30 (start of 30s), and only 3.7% (95% CI: 2.6–5.2%) remained underweight until the midpoint of their 30s (~age 34–35). By the endpoint of their 30s (~age 39), only 1.1% remained underweight (Table 3).

According to the results of the Kaplan–Meier analysis (Method 4), the probability of remaining underweight among women progressively decreased: 67.4% (95% CI: 65.4–69.3%) at 1 year, 37.9% (34.4–41.8%) at 5 years, and 25.2% (21.3–29.7%) at 10 years. The median time to the first transition was 2.5 years (95% CI: 2.2–2.9 years). The cumulative incidence of improvement reached 32.6% at 1 year, 62.1% at 5 years, and 74.8% at 10 years, with all individuals eventually experiencing at least one transition to normal weight by 21 years (Table 3).

Sensitivity analyses were performed to assess whether dichotomization at a BMI of 18.5 kg/m^2^ could account for the observed difference between short-term stability and continuous maintenance. When alternative thresholds (BMI < 18.3 and <18.7 kg/m^2^) were used, Method 1 (short-term year-to-year maintenance) remained high (74.55%, 76.94%, and 78.17% for 18.3, 18.5, and 18.7 kg/m^2^, respectively), whereas Method 4 (Kaplan–Meier continuous maintenance probability) remained stable at approximately 30% (30.30%, 29.72%, and 30.94%). The persistence of this discrepancy across thresholds suggests that the observed heterogeneity reflects intrinsic transition dynamics rather than a cutoff artifact.

Among women in their 20s, the short-term persistence rate estimated using Method 1 (78.1%) markedly exceeded the continuous maintenance probability estimated using the Kaplan–Meier analysis (Method 4: 47.1%), yielding a 31-percentage-point discrepancy. This divergence indicates that year-to-year stability does not equate to uninterrupted longitudinal persistence.

### 3.3. Bidirectional Transition Rates by Age

To quantify the dynamic interplay between improvement (underweight → normal) and reversion (normal → underweight), we estimated age-specific bidirectional transition rates among the cohort underweight in their 20s.

**Figure 3:**
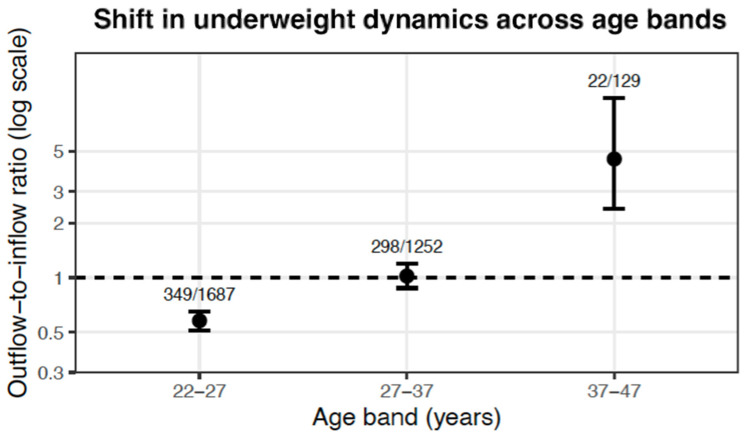
Structural shift in age-dependent underweight transition dynamics.

Age-stratified analyses of observation-based transition probabilities revealed a marked structural shift in underweight dynamics (Figure 3). Among women aged 22–27 years, the outflow-to-inflow ratio was 0.58 (95% bootstrap CI: 0.51–0.65), indicating inflow-dominant dynamics. This ratio approached equilibrium in the 27–37 age band (1.02, 0.88–1.19) and increased substantially after age 37 (4.54, 2.40–9.83), reflecting a reversal toward outflow-dominant transitions (Figure 3 and Table 4). Thus, the transition structure shifted progressively across age bands, crossing the equilibrium threshold (ratio = 1). These findings indicate that underweight transition dynamics are age-dependent and non-stationary rather than governed by a stable equilibrium process. Thus, underweight dynamics vary systematically across age bands, supporting a non-stationary transition framework.

Observation-based outflow-to-inflow ratios across age bands demonstrate a marked non-stationary transition structure. Among women, ratios increased progressively with age, crossing the equilibrium threshold (ratio = 1) and indicating a reversal from inflow-dominant to outflow-dominant dynamics. Error bars represent bootstrap 95% confidence intervals; event counts are shown to indicate sampling precision.

These bidirectional rates reveal age-dependent shifts in the balance between improvement and deterioration. The early 20s represent a high-risk period where weight cycling and new-onset underweight predominate, particularly among females. The dramatic increase in improvement-to-reversion ratios in the 37–47 age band for both sexes (>4:1) suggests that underweight becomes increasingly refractory with age—not due to inability to improve, but rather because the pool of individuals at risk of reverting from normal to underweight contracts substantially as weight stability increases with age. These results suggest that among underweight women in their twenties, their weight tends to repeatedly fluctuate between being underweight and normal weight, leading to the inference that the BMI distribution for underweight women likely peaks around 18.5.

### 3.4. BMI Threshold Clustering and Dynamic Fluctuation Patterns

#### 3.4.1. Participant Characteristics

**Figure 4:**
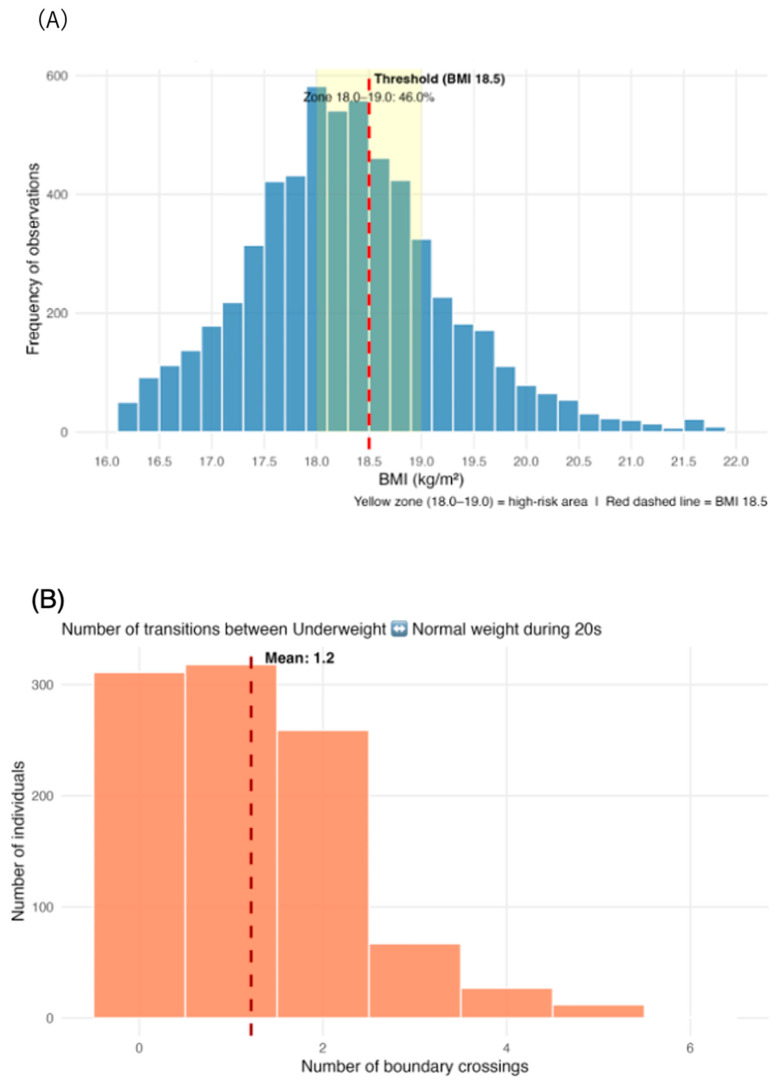

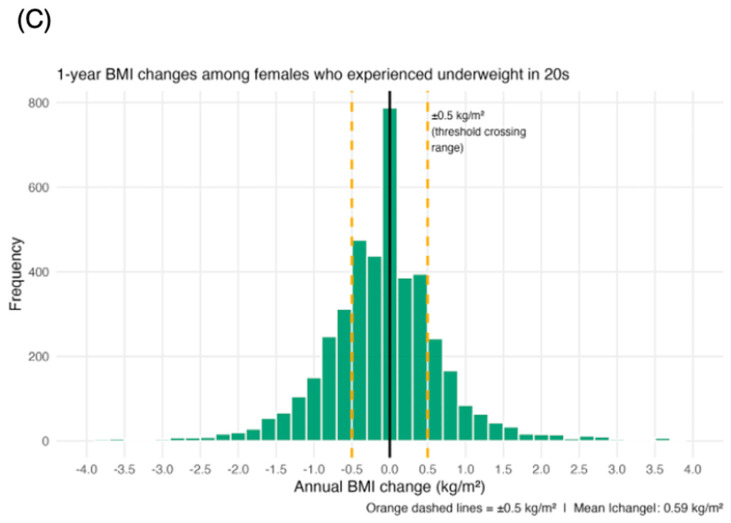
BMI Distribution Around the Underweight Threshold, Frequency of Boundary Crossings (BMI 18.5), and Annual BMI Change Distribution in underweight women. (**A**) Distribution of all BMI observations from women who experienced being underweight (BMI < 18.5 kg/m^2^) in their 20s (*n* = 883 women). The analysis includes all observations across the entire follow-up period (ages 20–60). The x-axis shows BMI (kg/m^2^) and the y-axis shows frequency of observations. Red dashed vertical line indicates the WHO underweight threshold (BMI 18.5 kg/m^2^). Yellow zone highlights the high-risk threshold region (BMI: 18.0–19.0 kg/m^2^) where frequent state transitions occur. The distribution reveals clustering around the BMI 18.5 cutoff, with approximately 46% of observations falling within the yellow threshold zone, demonstrating the dynamic nature of weight status near the diagnostic boundary. (**B**) Distribution of the number of transitions between underweight (BMI <18.5 kg/m^2^) and normal weight (BMI ≥ 18.5 kg/m^2^) per individual during the observation period. Based on 883 individuals with a mean of 6.1 observations each. The x-axis shows the number of boundary crossings during the 20s, and the y-axis shows the number of individuals. Red dashed vertical line indicates the mean value (1.2 crossings per person for both sexes). The distribution demonstrates high variability in weight status stability, with a substantial proportion of individuals (>50%) experiencing multiple state transitions. This “yo-yo” pattern indicates that underweight status is not a stable condition for many individuals, supporting the need for longitudinal rather than cross-sectional classification approaches. (**C**) Distribution of 1-year BMI changes among individuals who experienced underweight in their 20s. The x-axis shows annual BMI change (kg/m^2^/year) and the y-axis shows frequency. Solid black vertical line indicates zero change (no weight change). Orange dashed lines represent ±0.5 kg/m^2^ (typical margin for threshold crossing). Green shaded area highlights the range where BMI changes frequently exceed the threshold crossing margin. Mean absolute change: 0.59 kg/m^2^/year for both sexes. The distribution is approximately normal and centered near zero but with substantial tails indicating that many individuals experience annual changes >±0.5 kg/m^2^, which is sufficient to cross the BMI 18.5 threshold. This variability explains the high frequency of boundary crossings observed in Figure 4B.

The substantial discrepancy between Method 1 (78%) and Methods 2–4 (37–60%) suggested the presence of two heterogeneous subgroups: those oscillating around the threshold and those who were persistently underweight. We examined BMI distribution patterns, boundary-crossing frequency, and individual trajectories to investigate the mechanisms sustaining both the yo-yo phenomenon and long-term persistence (Figure 4). We investigated this hypothesis by examining BMI clustering and boundary-crossing patterns near the diagnostic cutoff (Figure 4A–C).

We performed a state transition analysis to characterize BMI dynamics across clinical thresholds. We calculated the empirical transition probabilities between consecutive annual observations and quantified the boundary crossing frequency. The underweight cohort in their 20s included 883 women, accounting for 5406 BMI observations from ages 20–29 (Table 5). The mean BMI of these women was 18.27 kg/m^2^.

#### 3.4.2. Threshold Zone Concentrations

A substantial proportion of observations clustered near the diagnostic cutoff BMI of 18.5 kg/m^2^ (Figure 4A). A total of 42.7% of the observations from women fell within the threshold zone (BMI of 18.0–19.0 kg/m^2^), representing nearly half of all underweight cohort measurements. This concentration was even more pronounced in the narrow zone (BMI of 18.25–18.75 kg/m^2^) with 22.1% of women (Table 5).

#### 3.4.3. Boundary Crossing Frequency

Approximately 70% of individuals crossed the BMI 18.5 kg/m^2^ boundary at least once during their twenties (69.4% women) (Figure 4B). Notably, 11.0% of women exhibited a “yo-yo pattern” (≥3 crossings), indicating repeated cycling between underweight and normal weight (Table 5).

#### 3.4.4. Annual Change in BMI

The mean absolute annual change in BMI was 0.58 kg/m^2^/year (SD 0.68) for women (Figure 4C, Table 5). A total of 44.7% of women’s transitions involved changes ≥0.5 kg/m^2^/year, suggesting that substantial fluctuations—sufficiently crossing the 18.5 kg/m^2^ BMI threshold within 1–2 years—were common. These findings align with the observed high frequency of boundary crossings.

### 3.5. Persistence Classification: Time-Weighted Persistence Patterns

#### 3.5.1. Validation of Persistence Categories: Mean BMI Differences (Sex-Stratified)

**Figure 5:**
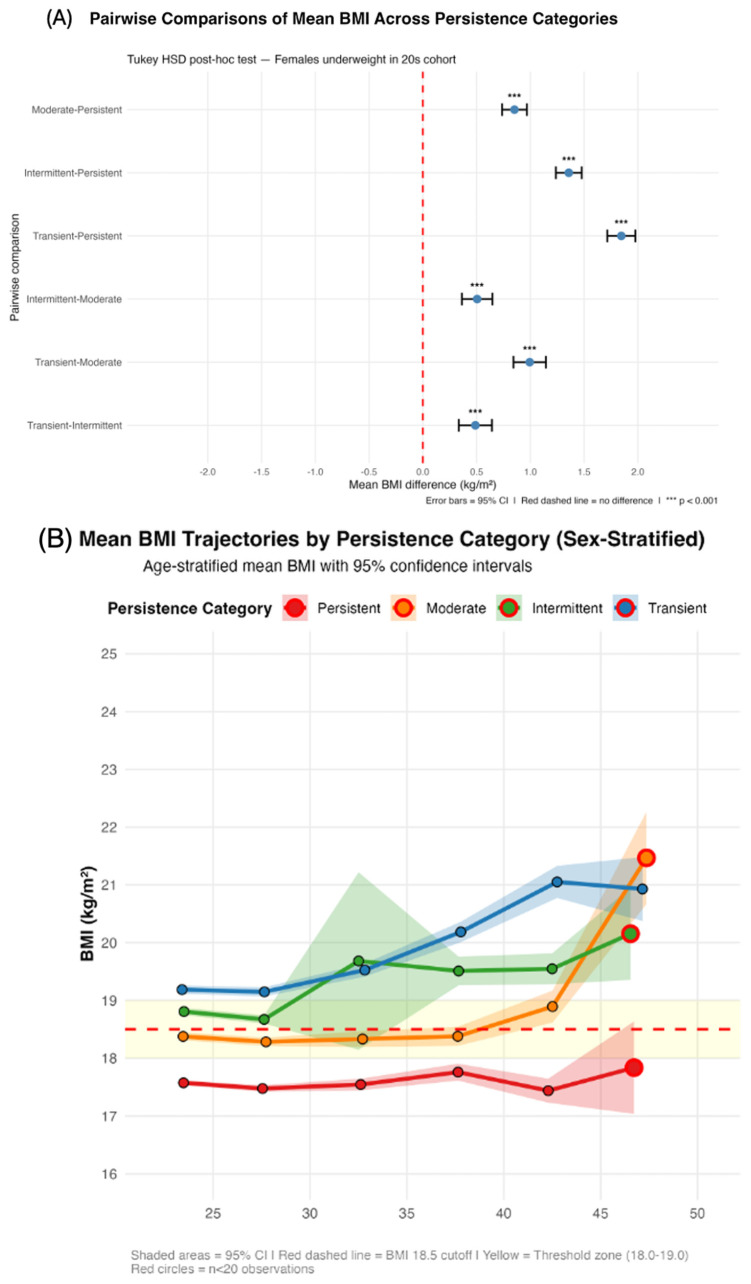

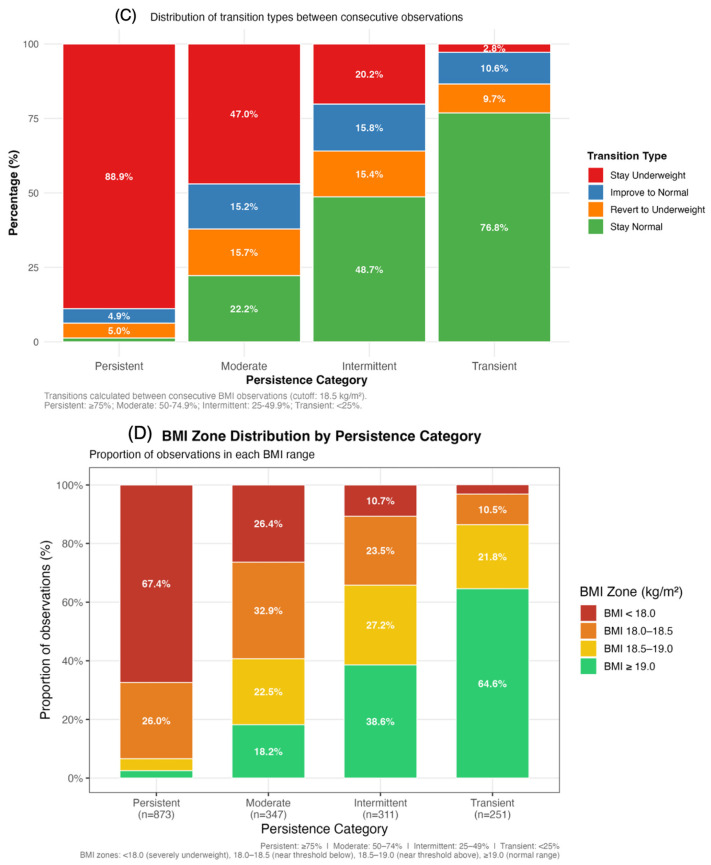
Persistence classification: time-weighted persistence patterns. (**A**) Tukey HSD post hoc pairwise comparisons of mean BMI differences between persistence categories, adjusted for age. Blue circles: significant (*p* < 0.05). Gray circles: not significant. Error bars: 95% CI. Red line: no difference. Largest difference: Transient vs. Persistent (Females: +1.8 kg/m^2^, 95% CI: 1.6–2.0). Results confirm time-weighted persistence successfully stratifies clinically meaningful BMI subgroups. *** *p* < 0.001. (**B**) Age-stratified mean BMI trajectories for four persistence categories (*N* = 883). Persistent (red, *n* = 389, 39.1%, ≥75% time underweight): stable low BMI ~17.5 kg/m^2^; 88.7% of consecutive observations remained underweight. Moderate (orange, *n* = 169, 17.0%, 50–74.9%) and Intermittent (green, *n* = 186, 18.7%, 25–49.9%): clustered around threshold zone (18.0–19.0 kg/m^2^) with ~30% bidirectional transitions. Transient (blue, *n* = 251, 25.2%, <25%): progressive recovery to normal weight (19.2→20.9–22.4 kg/m^2^); 76.6% maintained normal weight after recovery. Red dashed line: BMI 18.5 cutoff. Yellow zone: threshold region. Red circles: small sample (*n* < 20). Patterns similar between sexes (*p* = 0.613). (**C**) Distribution of transition types between consecutive BMI observations by persistence category (6035 observation pairs). Red: Stay Underweight (both <18.5). Blue: Improve to Normal (underweight → normal). Orange: Revert to Underweight (normal → underweight). Green: Stay Normal (both ≥18.5). Persistent group: 88.7% stayed underweight (stable chronic pattern). Transient group: 76.0–80.2% stayed normal (sustained recovery). Moderate/Intermittent: highest bidirectional instability, clustered around BMI 18.5 threshold. Patterns highly similar between sexes. (**D**) Stacked bars represent the proportion of all follow-up observations falling within each of four BMI zones: severely underweight (BMI < 18.0 kg/m^2^, dark red), near-threshold below (18.0–18.4 kg/m^2^, orange), near-threshold above (18.5–18.9 kg/m^2^, yellow), and normal range (≥19.0 kg/m^2^, green). Persistence categories were defined based on the proportion of total follow-up observations classified as underweight: Persistent (≥75%), Moderate (50–74%), Intermittent (25–49%), and Transient (<25%). Numbers in parentheses indicate the number of individuals in each category. Percentages ≥ 5% are shown within each segment.

The 883 individuals in the underweight cohort in the 20s age group were classified by the proportion of follow-up time spent with a BMI < 18.5 kg/m^2^. Four distinct subgroups emerged (Table 6, Figure 5A–D).

Among women, those who were persistently underweight (≥75%; *n* = 354, 40.1%) had a mean BMI of 17.54 ± 0.84 kg/m^2^. A total of 67.9% of the observations fell below the BMI of 18.0 kg/m^2^, which is substantially lower than the diagnostic cutoff (Table 6, Figure 5A–D). Only 29.2% clustered in the threshold zone (18.0–19.0%), predominantly in the lower range (24.7% in 18.0–18.5 vs. 4.2% in 18.5–19.0). Only 2.8% of the participants exceeded a BMI of 19.0 kg/m^2^. This reflects the chronic persistence of low body weight well below the diagnostic threshold.

Moderate (50–74%; *n* = 155, 17.6%) and intermittent (25–49%; *n* = 155, 17.6%) BMIs had mean values of 18.4 ± 1.07 and 19.0 ± 4.1 kg/m^2^, respectively, with the highest threshold zone concentrations (54.5% and 52.4%) (Table 6, Figure 5A–D). These subgroups exhibited a yo-yo pattern oscillating around a BMI of 18.5 kg/m^2^.

The transient (<25%; *n* = 219, 24.8%) group had a mean BMI 19.5 ± 1.30 kg/m^2^. A total of 62.3% of the participants had a BMI ≥ 19.0 kg/m^2^, and only 3.4% had a BMI less than 18.0 kg/m^2^. Despite similar threshold zone concentrations (33.4% vs. 28.9%), the observations were distributed in the upper range, indicating a transient, measurement-related underweight rather than a low BMI (Table 6, Figure 5A–D).

Statistical comparisons across the four persistence categories revealed highly significant differences in all the examined parameters. One-way ANOVA showed substantial variation in the mean BMI across groups (F(3897) = 250.43, *p* < 0.001) (Table 6). Post hoc pairwise comparisons via Tukey’s honest significant difference (HSD) test revealed significant differences between all category pairs (all *p* < 0.001; Figure 5A). The greatest difference in BMI was observed between the transient and persistent categories (mean difference = 1.83 kg/m^2^, 95% CI: 1.66–1.99, *p* < 0.001; Figure 5A), which was equivalent to approximately 4.5–5.5 kg in body weight for an individual with an average height of 160 cm. Other pairwise comparisons revealed a stepwise gradient: persistent vs. moderate (difference = 0.87 kg/m^2^, 95% CI: 0.68–1.06, *p* < 0.001), moderate vs. intermittent (difference = 0.51 kg/m^2^, 95% CI: 0.29–0.72, *p* < 0.001), and intermittent vs. transient (difference = 0.45 kg/m^2^, 95% CI: 0.25–0.65, *p* < 0.001) (Figure 5A).

These results confirm that the four persistence categories represent distinct, nonoverlapping BMI phenotypes, supporting the hypothesis of heterogeneous underweight persistence patterns.

Chi-square tests further indicate significant heterogeneity in the BMI distribution patterns (χ^2^(9) = 4161.33, *p* < 0.001) and boundary-crossing frequency of the yo-yo pattern (≥3) (χ^2^(3) = 146.71, *p* < 0.001) across the persistence categories (Table 6).

##### Threshold Zone Clustering

An analysis of the BMI distribution relative to the underweight threshold (18.5 kg/m^2^) revealed marked heterogeneity in the zone clustering patterns (Table 6). The threshold zone (BMI: 18.0–19.0 kg/m^2^, representing ±0.5 kg/m^2^ from the cutoff) contained 54.0% of the observations in the moderate group and 50.5% in the intermittent group, compared with 29.2% in the persistent group and 33.3% in the transient group (χ^2^(3) = 406.30, *p* < 0.001, Cramer’s V = 0.213) (Table 6).

The pronounced clustering in the moderate/intermittent groups suggests that these individuals spend the majority of their observation time in a BMI range where small physiological or measurement variations can alter their underweight classification, explaining the high frequency of boundary crossings (mean of 2.59–2.63 per person) and yo-yo patterns (42.6–43.2% prevalence) observed in these categories.

These findings provide robust statistical evidence for distinct subgroups within the underweight population that are characterized by fundamentally different persistence patterns and BMI trajectories.

#### 3.5.2. Identification of Distinct Subgroups by Time-Weighted Persistence

The time-weighted persistence analysis identified four distinct subgroups among women who were initially underweight in their 20s (Figure 5A, Table 6). The persistent category (*n* = 354, 40.1%) had a mean baseline BMI of 17.54 ± 0.84 kg/m^2^, with 68.3% of observations below the 18.0 kg/m^2^ threshold. The moderate category (*n* = 155, 17.6%) had a mean BMI of 18.40 ± 1.07 kg/m^2^, the intermittent category (*n* = 155, 17.6%) had a mean BMI of 18.97 ± 4.55 kg/m^2^, and the transient category (*n* = 219, 24.8%) had a mean BMI of 19.49 ± 1.30 kg/m^2^. All pairwise differences in the mean BMI were statistically significant (ANOVA F = 250.43, *p* < 0.001).

#### 3.5.3. Mean BMI Trajectories for Each Persistence Category (Sex-Stratified Analysis, Ages 20–50 Years)

The mean BMI trajectories differed markedly across the persistence categories (Figure 5B). The persistent group maintained a consistently low BMI throughout the observation period, with the mean BMI remaining below 18.0 kg/m^2^ across all age strata in women (range: 17.5–17.8 kg/m^2^). In contrast, the BMI of the transient group increased rapidly, crossing the underweight threshold (18.5 kg/m^2^) by age 30 in women (mean BMI: 19.8 kg/m^2^), ultimately reaching 21.5 kg/m^2^ (women) by age 50 (Table 6). The moderate and intermittent categories showed intermediate trajectories, with the mean BMI fluctuating around the 18.0–19.0 kg/m^2^ threshold zone throughout follow-up (Table 6). Notably, 54.5% of the observations in the moderate category and 52.4% of the observations in the intermittent category fell within this threshold zone (18.0–19.0 kg/m^2^), which were higher than the percentages of the persistent (12.3%) and transient (8.7%) categories.

#### 3.5.4. Annual State Transition Patterns for Each Persistence Category (Sex-Stratified Analysis, Ages 20–50+)

The distribution of year-to-year BMI changes pooled across all observation periods was analyzed. The state transition patterns differed substantially across the persistence categories (Figure 5C). The persistent group showed minimal state transitions, with 88.7% of women’s transitions being “stay underweight” and only 5.1% “improving to normal” (Figure 5C). The number of state transitions per individual was lowest in the persistent category (mean 0.69 ± 1.05 transitions), and only 5.4% of this group experienced ≥3 transitions during follow-up (Figure 5C). In contrast, the moderate and intermittent categories showed frequent bidirectional fluctuations between the underweight and normal weight states. Among women in the moderate category, 22.5% of the transitions were “improving to normal,” 16.4% were “racing to underweight,” and 14.9% were “stay underweight” (Figure 5C). Women in the intermittent category showed similar patterns (48.0% “stay normal,” 18.3% “improving to normal,” 16.3% “racing to underweight”) (Figure 5C). Notably, 42.6% of women in the moderate group and 43.2% of women in the intermittent group experienced ≥3 state transitions, which was significantly greater than that of the persistent group (5.4%, *p* < 0.001). The transient category was characterized predominantly by a stable normal weight status after an initial improvement, with 76.0% of women’s transitions being “stay normal” and only 10.3% being “racing to underweight”.

Similarly, Figure 5D shows the distribution of BMI zones by persistence category among women. In the Persistent group, 67.4% fell into the BMI < 18.0 (severely underweight) category, and 26.0% had a BMI of 18.0–18.5 (just below the threshold). In the Moderate group, the percentage with BMI < 18.0 decreased to 32.9%, while those with BMI ≥ 19.0 (normal range) accounted for 18.2%. In the Transient group, 64.6% had BMI ≥ 19.0, and only 10.5% had BMI < 18.0. There was a clear gradient: as the persistence category decreased, the proportion of severely underweight individuals declined, while the proportion in the normal BMI range increased (Figure 5D).

Thus, the discrepancy between short-term (Method 1: 78%) and time-weighted (Method 4: 47%) persistence estimates arise from two complementary mechanisms: the “yo-yo effect” and threshold clustering and phenotypic heterogeneity. These findings demonstrate that the methodological choice is not merely a technical consideration but fundamentally shapes the clinical and epidemiological interpretation of the prevalence and persistence of underweight. By conflating transient boundary fluctuations with true long-term persistence, cross-sectional surveys (analogous to Method 1) may substantially overestimate the burden of chronic underweight.

Together, the discrepancy between conditional short-term stability and sustained longitudinal maintenance suggests that the cross-sectional prevalence may not fully represent within-individual BMI dynamics (i.e., the assumption of ergodicity may not fully apply). Notably, in the underweight cohort in their 20s, the gap between estimates derived from Method 1 and Method 4 was approximately 31 percentage points, highlighting substantial divergence between year-to-year stability and continuous maintenance.

## 4. Discussion

In this study, short-term conditional persistence (Method 1: 78% in women and 73% in men) systematically overestimated long-term maintenance (Methods 2–4: 37–60%) by up to 42 percentage points. Observation-based outflow-to-inflow ratios across age bands demonstrated a marked non-stationary transition structure, with ratios increasing progressively with age and indicating a shift from inflow-dominant (normal-to-underweight) to outflow-dominant (underweight-to-normal) dynamics. Exploratory analysis of the BMI distribution and boundary-crossing patterns revealed bimodal clustering and heterogeneous temporal dynamics, leading us to develop a time-weighted persistence classification that identified four subgroups with distinct BMI trajectory patterns: 40% chronic persistent (mean BMI 17.54 kg/m^2^), 35% yo-yo fluctuating (repeatedly crossing the 18.5 kg/m^2^ threshold and accounting for the observed discrepancy), and 25% transient. Without this multimethod integration, the yo-yo group would remain hidden, resulting in systematic overestimation of the long-term underweight burden. This study therefore provides an important clue for distinguishing between persistently underweight individuals and those whose underweight status does not persist, and is one of the few investigations that help clarify the current, largely unexplored situation of underweight in Japan.

This study demonstrated that underweight maintenance in young adults is not a unitary condition but comprises distinct temporal phenotypes. While short-term conditional persistence (Method 1) suggested high annual stability (78.1% in women and 73.2% in men), continuous maintenance estimated by the Kaplan–Meier analysis (Method 4) was substantially lower, producing a 31-percentage-point discrepancy in women (78.1% vs. 47.1%). This gap persisted across alternative BMI thresholds (18.3 and 18.7 kg/m^2^), indicating not solely explained by the cutoff. Observation-based outflow-to-inflow ratios across age bands demonstrate that ratios increased progressively with age, indicating a reversal from inflow (normal to underweight)-dominant to outflow (underweight to normal)-dominant dynamics. Moreover, exploratory analyses revealed pronounced clustering within the 18.0–19.0 kg/m^2^ range and frequent threshold crossings, with approximately 70% of individuals crossing the 18.5 kg/m^2^ BMI boundary at least once during follow-up. These patterns indicate that a substantial fraction of the cohort resides near the diagnostic boundary. Based on the above, it is believed that there is a shift from an influx into underweight at younger ages to an outflow into normal weight as age increases, and that the BMI of people who were diagnosed as underweight even once in their 20s is concentrated around the BMI range of 18.0–19.0, resulting in a discrepancy between the short-term underweight maintenance rate and the long-term underweight maintenance rate.

Time-weighted phenotyping further distinguished four groups: persistent (40.1%, mean BMI of 17.54 kg/m^2^), moderate (17.6%), intermittent (17.6%), and transient (24.8%). The moderate and intermittent groups (35.2%) formed a boundary-crossing “yo-yo” phenotype characterized by repeated transitions and residence near the threshold zone. In contrast, the persistent phenotype maintained a structurally low BMI with minimal crossing. Importantly, the marked divergence between short-term and continuous estimates suggests that population-level stability does not reflect within-individual trajectories, suggesting heterogeneity between short-term and long-term patterns [23,24,25]. These findings explain the 31-point discrepancy: boundary-crossing individuals appear stable in conditional year-to-year analyses but do not sustain an uninterrupted underweight status over time. Without longitudinal integration, this subgroup would remain obscured.

The 31-percentage-point gap between short-term conditional stability and sustained longitudinal maintenance may have implications for future clinical research. Cross-sectional or year-to-year assessments (Method 1) classify many individuals as “persistent,” whereas time-weighted and survival-based approaches suggest that only a minority maintain continuous underweight over time. Reliance on short-term stability may therefore overestimate the burden of chronic underweight. This discrepancy appears to reflect a boundary-crossing subgroup whose BMI fluctuates around the diagnostic threshold: such individuals may appear stable in annual comparisons but do not sustain continuous underweight exposure over time. Without a trajectory-based evaluation, they may be misclassified as being persistently underweight despite only being intermittently underweight. Conversely, another subgroup maintained a BMI consistently below the threshold, suggesting more sustained exposure to low body weight. For this subgroup, cumulative exposure rather than momentary classification may be relevant. Persistent low body weight may be relevant to fracture risk in postmenopausal women, as low BMI is a well-established risk factor for fragility fractures in this population [26,27,28]. Recent studies further suggest that repeated or cumulative exposure to underweight is associated with higher hip fracture risk [29,30]. However, the duration or cumulative exposure threshold required to materially increase fracture risk remains unclear. A similar cumulative exposure concept has been proposed in familial hypercholesterolemia, in which LDL-cholesterol burden over time is considered important in the development of myocardial infarction, although the precise threshold remains uncertain, particularly in FH populations [31,32]. Because fracture, metabolic, reproductive, and mortality outcomes were not assessed in the present study, these considerations should be regarded as hypothesis-generating. Accordingly, time-weighted persistence may be useful as a descriptive framework for future studies on risk stratification, pending validation against clinically meaningful outcomes. In support of this interpretation, a study of 20-year-old Japanese female university students identified groups whose BMI standard deviation score remained consistently low from childhood and groups whose BMI standard deviation score declined after adolescence [17]. Taken together, these findings suggest that trajectory-based approaches may complement cross-sectional categorization in the assessment of young adults with low BMI.

Importantly, our study does not include fracture, reproductive, metabolic, or mortality outcomes. Therefore, we cannot determine whether the persistent subgroup has a greater clinical risk. Our findings should not be interpreted as direct evidence of adverse health consequences. Rather, we demonstrate structural heterogeneity in cumulative exposure to a low BMI. Individuals classified as underweight at a single time point differ markedly from young adults who spend time with a BMI less than the diagnostic threshold. Yang et al. demonstrated six trajectories among 29,881 adults: lower-normal stable, higher-normal stable, normal → overweight, chronic borderline obesity, normal → class I obesity, and overweight → class II obesity. They compared the mortality risk for each class and reported that the trajectory of maintaining borderline obesity over the long term is associated with a high risk [33]. In a study involving 80,000 people, weight trajectories over approximately five years (stable, increasing, decreasing, and weight cycling) were classified, and the risks of 10 diseases, including heart failure and diabetes, were evaluated. Both weight cycling and sustained weight gain were reported to be associated with higher risks compared to stable weight cycling [34]. Among 30,581 participants in the Danish Diet, Cancer, and Health-Next Generations cohort, four BMI trajectories (stable low, moderate increase, steeper increase, and early high BMI) were identified over a 50-year span from adolescence to middle and older age. Higher and more rapidly increasing trajectories were associated with worse cardiometabolic risk factors such as blood glucose levels, lipid levels, and blood pressure [35]. This distinction may be relevant for future outcome studies, particularly those examining cumulative biological effects such as bone mass accrual. Accordingly, our results are best interpreted as a framework for describing heterogeneity rather than as proof of risk. Based on the framework of the low body weight subgroup used this time, it will be necessary in the future to link low-body-weight individuals with clinical markers such as fracture risk.

This study has several limitations. First, it was conducted at a single center, potentially limiting generalizability. Second, the number of male participants was too small to allow for meaningful analysis, and therefore men could not be included in the present study. In addition, the number of participants in their 50s was insufficient, precluding analysis of older age groups. Accordingly, our findings should be interpreted primarily within the female population and age range available for analysis. Future studies should include both men and older individuals to determine whether the heterogeneity of BMI trajectories observed here can be generalized across sex and broader age groups. Third, we lacked data on behavioral, hormonal, and metabolic factors that could explain the differences in the trajectories. Fourth, the measurement variability near the 18.5 kg/m^2^ BMI threshold may contribute to boundary crossings; however, the consistent bimodal distribution and subgroup structure argue against pure measurement artifacts. Fourth, we did not assess clinical outcomes (e.g., fracture, reproductive outcomes, and mortality). Therefore, our findings describe structural heterogeneity in BMI dynamics rather than direct health risks. Finally, since this study was conducted at a single institution, it is unclear whether these findings are applicable nationwide in Japan. In addition, since the number of men is overwhelmingly smaller compared to women, there is a risk that the analysis results may become unstable. We plan to use health insurance association data covering several million people to clarify whether the results of this study are representative of the entire country.

## 5. Conclusions

In this cohort, underweight in young adulthood appeared to be heterogeneous rather than uniform. Across the four analytical approaches, short-term conditional persistence yielded substantially higher estimates than sustained longitudinal maintenance, with an absolute difference of approximately 31 percentage points. This discrepancy remained broadly similar across alternative BMI thresholds, suggesting that it was not solely attributable to the conventional cutoff of 18.5 kg/m^2^. Using a time-weighted classification, we identified four subgroups with different patterns of underweight persistence, including chronically low, fluctuating, and transient BMI patterns. The discrepancy between short-term and long-term persistence appeared to be driven largely by individuals with repeated transitions across the BMI threshold.

These findings demonstrate heterogeneity in BMI trajectories within the studied population but they do not constitute definitive evidence for a new clinical definition of underweight. Rather, they suggest that cross-sectional BMI alone may not fully capture longitudinal variation in underweight persistence. Because clinical outcomes were not assessed, the present results should be interpreted as descriptive and hypothesis-generating. External validation and outcome-based studies are needed before trajectory-based classifications can inform risk stratification or clinical thresholds.

## Figures and Tables

**Table 1:** Baseline Characteristics of female employees.

	Level	Female
*N*		4328
Age (years)		27.84 (8.84) ^a^
Height (cm)		157.79 (5.24) ^a^
BW (kg)		52.18 (7.61) ^a^
BMI (kg/m^2^)		20.94 (2.78) ^a^
Age group *N* (%)	20s	3085 (71.3)
	30s	609 (14.1)
	40s	491 (11.3)
	50s	143 (3.3)
BMI state (%)	Normal weight	3394 (78.4)
	Overweight	322 (7.4)
	Underweight	612 (14.1)
Follow-up duration (years)		8.2 (5.5) ^a^
Follow-up duration (years)		6.0 (4.0–11.0) ^b^

Data are represented as mean (SD) ^a^ and median (IQR) ^b^.

**Table 4:** Bidirectional Transition Rates by Age.

Age_Band (Years)	Ratio_With_95%CI	Inflow_Events	Outflow_Events
22–27	0.58 (0.51–0.65)	427/1195	349/1687
27–37	1.02 (0.88–1.19)	216/928	298/1252
37–47	4.54 (2.40–9.83)	13/346	22/129

Inflow = Normal → UW/Normal observations, Outflow = UW → nonUW/UW observations, Ratio = outflow/inflow (bootstrap 95% CI).

**Table 5:** BMI Threshold Clustering and Dynamic Fluctuation Patterns.

Metric	Female
Mean BMI (kg/m^2^)	18.27 (0.87) ^a^
Threshold Zone 18.0–19.0 (%)	42.71%
Narrow Zone 18.25–18.75 (%)	22.12%
Mean boundary crossings	1.23 (1.11) ^a^
Yo-yo pattern ≥3 (%)	10.87%
Annual BMI change (kg/m^2^/year)	0.58 (0.69) ^a^
Changes ≥0.5 kg/m^2^ (%)	44.67%

Drawing on approaches used in weight status transition studies [20,21], we quantified BMI clustering around the diagnostic threshold and characterized dynamic weight fluctuation patterns. Similar to multi-state Markov models that estimate transition intensities and sojourn times [20,22], we analyzed boundary-crossing frequency—defined as transitions across the BMI 18.5 kg/m^2^ threshold between consecutive observations. However, we extended this framework by: 1. Threshold Zone Concentration: Explicitly quantifying the proportion of observations within a ±0.5 kg/m^2^ band around the clinical cutoff (BMI 18.0–19.0 kg/m^2^), capturing borderline underweight status where measurement error and biological fluctuation may result in frequent state transitions. 2. Recurrent Fluctuation Patterns: Defining individuals with ≥3 boundary crossings as exhibiting “recurrent fluctuation patterns,” analogous to weight cycling observed in obesity research [21]. 3. Annual Change Magnitude: Classifying annual BMI changes ≥ 0.5 kg/m^2^/year as “substantial fluctuations,” as this magnitude can easily facilitate boundary crossings across the 1.0 kg/m^2^ threshold zone width. ^a^ Dara represented as mean (SD). Note: Values are presented as mean ± SD or percentages. Threshold Zone: BMI 18.0–19.0 kg/m^2^ (±0.5 kg/m^2^ from the underweight cutoff of 18.5 kg/m^2^). Narrow Zone: BMI 18.25–18.75 kg/m^2^ (±0.25 kg/m^2^ from the cutoff). Boundary crossing: transition between BMI <18.5 kg/m^2^ and BMI ≥18.5 kg/m^2^. Yo-yo pattern: ≥3 boundary crossings during follow-up. Annual BMI change: absolute value of 1-year BMI change.

**Table 6:** Persistence classification: time-weighted persistence patterns.

Characteristic	Persistent (≥75%) ^a^	Moderate (50–74.9%) ^a^	Intermittent (25–49.9%) ^a^	Transient (<25%) ^a^	Statistical Test
*n* individuals (*n* = 883), %	354 (40.0%)	155 (17.6%)	155 (17.6%)	219 (24.8%)	
Total observation	2779	1441	1404	2207	
BMI, kg/m^2 b^	17.54 (0.84)	18.40 (1.07)	18.97 (4.55)	19.49 (1.30)	F(3879) = 250.43, *p* < 0.001 ^e^
95% CI ^c^	17.45–17.63	18.23–18.57	18.25–19.69	19.32–19.66	
BMI distribution, %					χ^2^(9) = 4161.33, *p* < 0.001 ^d^
<18.0 kg/m^2^	68.3	28.3	10.4	3.4	
18.0–18.5 kg/m^2^	24.7	32.6	24.8	10.9	
18.5–19.0 kg/m^2^	4.2	21.9	27.6	23.4	
≥19.0 kg/m^2^	2.8	17.2	37.2	62.3	
Threshold zone (18.0–19.0), %	28.9	54.5	52.4	34.3	χ^2^(3) = 389.53, *p* < 0.001 ^e^
Narrow Zone (18.25–18.75 (%))	12.2	29.1	31.4	18.6	χ^2^(3) = 289.51, *p* < 0.001 ^e^
Boundary-crossing patterns					
Crossings (number) ^a^	0.69 (1.05)	2.59 (1.80)	2.63 (1.52)	1.90 (0.98)	H(3) = 338.98, *p* < 0.001 ^f^
≥1 crossing, %	40.7	100	100	99.5	χ^2^(3) = 464.68, *p* < 0.001 ^g^
Yo-yo pattern (≥3), %	5.4	42.6	43.2	15.1	χ^2^(3) = 146.71, *p* < 0.001 ^h^

Footnotes: ^a^ Time-weighted persistence category defined as percentage of follow-up time spent in underweight state (BMI <18.5 kg/m^2^): Persistent: ≥75%; Moderate: 50–74.9%; Intermittent: 25–49.9%; Transient: <25%. ^b^ One-way ANOVA testing mean BMI differences across persistence categories within each sex group. ^c^ 95% confidence intervals calculated using standard error of the mean: CI = mean ± 1.96 × (SD/√*n*). For observation-level data, *n* = total observations. ^d^ Chi-square test for BMI distribution across four BMI zones by persistence category. ^e^ Chi-square test for threshold zone concentration (BMI 18.0–19.0 kg/m^2^) across persistence categories. This high-risk zone showed highest concentration in Moderate/Intermittent groups, suggesting measurement artifact effects near the underweight cutoff. ^f^ Kruskal–Wallis test for differences in number of boundary crossings (transitions between underweight [BMI <18.5] and normal weight [BMI ≥18.5]) across persistence categories. ^g^ Chi-square test for proportion with at least one boundary crossing across persistence categories. ^h^ Chi-square test for yo-yo pattern (≥3 boundary crossings) prevalence across persistence categories.

## Data Availability

The datasets presented in this article are not readily available because the data are part of an ongoing study. Requests to access the datasets should be directed to the correspondence author.
